# UbiNet: an online resource for exploring the functional associations and regulatory networks of protein ubiquitylation

**DOI:** 10.1093/database/baw054

**Published:** 2016-04-25

**Authors:** Van-Nui Nguyen, Kai-Yao Huang, Julia Tzu-Ya Weng, K. Robert Lai, Tzong-Yi Lee

**Affiliations:** ^1^Department of Computer Science and Engineering, Yuan Ze University, Taoyuan, 320, Taiwan; ^2^University of Information and Communication Technology, Thai Nguyen University, Vietnam and; ^3^Innovation Center for Big Data and Digital Convergence, Yuan Ze University, 320, Taiwan

## Abstract

Protein ubiquitylation catalyzed by E3 ubiquitin ligases are crucial in the regulation of many cellular processes. Owing to the high throughput of mass spectrometry-based proteomics, a number of methods have been developed for the experimental determination of ubiquitylation sites, leading to a large collection of ubiquitylation data. However, there exist no resources for the exploration of E3-ligase-associated regulatory networks of for ubiquitylated proteins in humans. Therefore, the UbiNet database was developed to provide a full investigation of protein ubiquitylation networks by incorporating experimentally verified E3 ligases, ubiquitylated substrates and protein–protein interactions (PPIs). To date, UbiNet has accumulated 43 948 experimentally verified ubiquitylation sites from 14 692 ubiquitylated proteins of humans. Additionally, we have manually curated 499 E3 ligases as well as two E1 activating and 46 E2 conjugating enzymes. To delineate the regulatory networks among E3 ligases and ubiquitylated proteins, a total of 430 530 PPIs were integrated into UbiNet for the exploration of ubiquitylation networks with an interactive network viewer. A case study demonstrated that UbiNet was able to decipher a scheme for the ubiquitylation of tumor proteins p63 and p73 that is consistent with their functions. Although the essential role of Mdm2 in p53 regulation is well studied, UbiNet revealed that Mdm2 and additional E3 ligases might be implicated in the regulation of other tumor proteins by protein ubiquitylation. Moreover, UbiNet could identify potential substrates for a specific E3 ligase based on PPIs and substrate motifs. With limited knowledge about the mechanisms through which ubiquitylated proteins are regulated by E3 ligases, UbiNet offers users an effective means for conducting preliminary analyses of protein ubiquitylation. The UbiNet database is now freely accessible via http://csb.cse.yzu.edu.tw/UbiNet/. The content is regularly updated with the literature and newly released data.

**Database URL:** http://csb.cse.yzu.edu.tw/UbiNet/.

## Introduction

Protein ubiquitylation, involving ubiquitin conjugation at a target lysine residue, is carried out through a series of enzymatic activities such as E1 activation, E2 conjugation and E3 ligation (1), as illustrated in Supplementary Figure S1. To date, high-throughput mass spectrometry (MS)-based proteomics approaches have facilitated the characterization of many substrate proteins with ubiquitylation sites (2–5). In particular, ubiquitylation of lysine residues was found to function as a crucial modulator in transcriptional regulation, signal transduction, development, apoptosis, endocytosis, cell proliferation and cancers (6–8). In protein ubiquitylation, E3 ligases are responsible for recognizing substrate sites for ubiquitin-mediated protein degradation (9). An E3 ligase could regulate and recognize multiple substrates in various functional networks; alternatively, each substrate may be regulated by multiple E3s (10). These relationships could be organized into a network of E3-specific regulatory activities against multiple cellular pathways. As a result, complex cellular processes could be characterized comprehensively with the integration of an E3-specific functional regulatory network by investigating the functional specificity of E3-specific substrates along with their networks (11).

As E3 ubiquitin ligases are important in protein regulation, many studies attempted to characterize E3 structures, examine E3-mediated regulatory networks and investigated E3-related diseases (12–15, 17). E3 ligases can be categorized based on their catalytic mechanisms. In general, E3 ligases involved in ubiquitylation can be classified into three classes, including the HECT homologous to E6-AP C-terminus (HECT), really interesting new gene (RING) and RINGbetween-RING (RBR) types (16). RING-type (really interesting new gene) E3s facilitate the transfer of ubiquitin from an E2 enzyme to the substrate, establishing binding between the E2∼Ub thioester and the substrate; while E3s belonging to the HECT-type (homologous to E6-AP C-terminus) and RBR-type (RING-between-RING) function by transferring ubiquitin from an E2 to the cysteine containing active site in an E3 enzyme, and subsequently to the substrate.

The significance of E3-mediated ubiquitylation is further underscored by their association with diseases (18). Indeed, several E3 ligases, such as Mdm2/Hdm2, Inhibitor of Apoptosis/ or Apoptosis inhibitor (IAPs) and Skp, Cullin, F-box containing complex (SCF), are over-expressed in many human cancers, and because evidence suggests that the inhibition of these enzymes may lead to growth suppression or apoptosis (19). Therefore, understanding of the mechanisms and interaction networks of E3 ligases may facilitate the development of more efficient cancer treatments.

The biological significance of E3 ligases in cellular processes has spurred efforts in the bioinformatics domain. For instance, E3Net (10) has presented a collection of 1671 E3-substrate relations between 493 E3s and 1277 substrates in 42 organisms. Additionally, Sakiyama *et al.* (20) built a database of proteins involved in the ubiquitin signaling cascade, and analysed their sequence similarities, domains and distributions across different species. Despite an increasing interest in ubiquitylated proteins and E3 ligases, there is a lack of resources dedicated to mapping the regulatory networks of E3 ligases for large-scale ubiquitylation data. Therefore, the UbiNet database was developed to provide an interactive network viewer for discovering protein ubiquitylation networks. To enable the comprehensive investigation of regulatory networks among E3 ligases and ubiquitylated proteins, metabolic pathways and protein–protein interactions (PPIs) were incorporated to explore protein ubiquitylation networks. Moreover, in order to facilitate the study of protein ubiquitylation and their functions, a web interface was developed for users to search for their proteins of interest. Published literature information related to E3 ligases, ubiquitylated proteins, ubiquitylation sites and PPIs are also provided in this online resource. Finally, case studies demonstrated that UbiNet could help users identify E3 ligase-mediated ubiquitylation networks and their biological roles. Within the scope of our current knowledge about the relationships among E3 ligases and ubiquitylated proteins, UbiNet provides potential E3 ligases for ubiquitylated proteins based on PPI information and substrate site specificities.

## Materials and Methods

### Database construction of UbiNet

**Figure 1.**
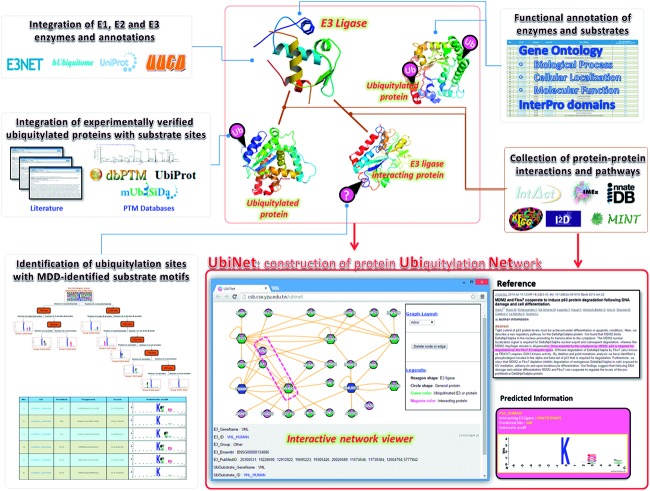
Flowchart for the construction of the UbiNet system.

Figure 1 presents the flowchart for the construction of UbiNet, including the database construction of E3 ligases and ubiquitylated proteins, functional and structural analyses, as well as the building of regulatory networks and identification of ubiquitylation sites with substrate motifs. In this study, experimentally verified data of human E1 activating enzymes, E2 conjugating enzymes and E3 ubiquitin ligases were manually curated from various databases. In total, two distinct proteins for E1 activating enzymes were extracted from UUCD-Version 1.0 (21). The number of distinct E3 and E2 enzymes, as well as the sources from which they were obtained, are listed in Supplementary Table S1. In total, 499 non-redundant E3 ubiquitin ligases and their biological functions were manually curated from E3Net, UUCD (21), hUbiquitome (22) and UniProtKB (23). Additionally, 46 non-redundant E2 conjugating enzymes were manually curated from UUCD, hUbiquitome and UniProtKB. These experimentally verified data were generated by a variety of methods, including protein interaction assays, mutation assays, substrate degradation or stability detection, immunoblots and mass spectrometry (22), as well as other approaches specified in the associated publications on the UniProtKB site (23).

Ubiquitylated proteins in the UbiNet database were extracted mainly from research articles through a search on the PubMed database using ‘ubiquitylated’, ‘ubiquitylation’, ‘ubiquitinated’ or ‘ubiquitination’ as the search keywords. The matched full-text research articles were subsequently downloaded. Then, a text-mining system was developed to select articles that potentially described site-specific information of ubiquitylation sites. Finally, to ensure the precise extraction of information regarding ubiquitylated peptides along with the modified lysine residues, all selected articles were manually reviewed. Also, experimentally verified ubiquitylation sites in dbPTM (24–26) were integrated into UbiNet with the corresponding literature references. After the removal of redundant data, a total of 43 948 ubiquitylated lysines were obtained from 14 692 human ubiquitylated proteins, which were supported by 464 literature articles.

### Functional and structural analyses of E3 ligases and ubiquitylated proteins

In order to ensure the comprehensive functional and structural annotations of proteins relevant to ubiquitylation, information from various biological databases, such as Gene Ontology (GO) (27), InterPro (28), Protein Data Bank (PDB) (29), as well as Kyoto Encyclopedia of Genes and Genomes (KEGG) Diseases and Pathways (30), was incorporated into UbiNet. The biological importance of ubiquitylated proteins were categorized based on their annotated molecular functions, biological processes and cellular components in GO. InterPro, which was developed initially as a means of rationalizing the complementary efforts of the PROSITE (31), PRINTS (32), Pfam (33) and ProDom (34) databases, is an integrated resource that provides essential information such as protein families, domains and functional sites. It has been reported that ubiquitylation regulates the degradation of proteins, coordinates protein cellular localizations and modulates PPIs. (35–37). Thus, functional domain information could be utilized to infer the functional roles of ubiquitylation sites located in a specific protein domain. Recently, the importance of protein ubiquitylation in the development and progression of several diseases and disorders is becoming recognized, and the molecules involved in these processes have been used as therapeutic agents for slowing down disease progression (12, 38–40). Accordingly, the disease annotations from KEGG Disease Database (41), Online Mendelian Inheritance in Man (42) and Human Protein Reference Database (43) were utilized to provide disease associated information for ubiquitylated proteins.

With the advancement in high-throughput MS-based proteomic techniques, the growing number of experimentally confirmed PTM Post-Translational Modification sites has prompted an increasing interest in the structural features of the substrate sites (44, 45). With limited protein structures containing the covalent attachment of ubiquitin to lysine (K) residues, all of the experimentally verified ubiquitylation sites were mapped to the protein entries of PDB (46) by Basic Local Alignment Search Tool with 100% sequence identity. DSSP (47) was then utilized to calculate the surface solvent accessibility and standardize the secondary structure of PDB entries with the mapped ubiquitylation substrate sites. Following previous studies investigating the structural characteristics of PTMs (45, 48, 49) in proteins without known tertiary structures, two effective tools, RVP-net (50) (an online program for the prediction of real valued solvent accessibility) and PSIPRED (51) (A server for protein structure prediction), were used to predict the solvent accessibility and secondary structure, respectively. To facilitate the structural investigation of substrate sites on ubiquitylated proteins, the solvent accessibility and secondary structure of a protein, as well as the ubiquitylation sites, were graphically represented using the PHP GD library. Moreover, the tertiary structures of ubiquitylated proteins and ubiquitylation sites were visualized using the Jmol program (52).

### Construction of regulatory networks between E3 ligases and ubiquitylated proteins

An increasing number of studies have suggested that protein ubiquitylation plays a crucial role in the regulation of biological processes. Therefore, we reasoned that the integration of experimentally validated E3 ubiquitin ligases would help provide a foundation for exploring ubiquitylation regulatory networks in mammals. In an attempt to facilitate the comprehensive investigation of the regulatory relationships among E3 ligases and substrates (ubiquitylated proteins), metabolic pathways and PPIs were incorporated for the exploration of protein ubiquitylation networks. The human metabolic pathways followed the annotations in KEGG (53). For experimentally verified interactions among proteins, over ten PPI databases (as listed in Supplementary Table S2) have been utilized. In addition to physical interactions, the STRING database also consists of predicted functional associations among proteins (co-regulation in curated pathways, co-occurrence in literature abstracts, mRNA co-expression and genomic context), where each connection was assigned a confidence score (54). In order to formalize the regulatory network between E3 ligases and substrates, a protein ubiquitylation network was visualized as a directed graph *G* = (*V*, *E*), where *x*, *y* ∈ *V* and (*x*, *y*) ∈ *E*. Here, *x* and *y* represent the E3 ligases and substrate proteins, respectively, and (*x*, *y*) ∈ *E* denotes protein ubiquitylation when E3 ligase *x* recognizes a specific substrate *y*. Note, however, the number of substrates that are known to be recognized by E3 ligases for ubiquitylation is very limited. Thus, (*x*, *y*) could also be seen as a type of interaction between E3 ligase *x* and ubiquitylated protein *y*. In this work, human proteins were symbolized by *V*, while experimentally verified PPIs were represented by *E*. Finally, Cytoscape (55) was utilized to design an interactive viewer for exploring E3-substrate ubiquitylation networks and the associated ubiquitylation sites.

### Identification of protein ubiquitylation sites with substrate motifs

Due to the time-consuming and labor-intensive lab work for identifying site-specific ubiquitylated peptides, a biologist, restricted by time and budget, may only be able to conclude that a protein can be ubiquitylated, but the exact ubiquitin-conjugation site remains unknown. Hence, an effective prediction method can pinpoint the potential ubiquitylation sites, helping a biologist work more efficiently. With reference to a previous prediction method for identifying ubiquitylation sites with various substrate site specificities (2), this work focused on characterizing the substrate motifs of human ubiquitylated sites to discover ubiquitin conjugation sites on the substrates. To overcome the difficulty of uncovering conserved motifs from large-scale ubiquitylation data, maximal dependence decomposition (MDD) (56) was used to identify the substrate motifs for protein ubiquitylation sites collected in UbiNet. The MDD method has demonstrated its effectiveness in identifying the substrate motifs of plant and virus phosphorylation (57, 58), acetylation (59), O-GlcNAcylation (60), *S*-glutathionylation (61), *S*-nitrosylation (62), *S*-sulfenylation (63), as well as protein ubiquitylation sites (2). MDD exploits an iteratively statistical method to discover conserved motifs from a group of aligned signal sequences. MDD can group a set of aligned signal sequences to moderate a large group into subgroups that capture the most significant dependencies between positions. As presented in Supplementary Figure S2, MDD adopts the chi-square test χ2 (*A*_i_, *A*_j_) to iteratively evaluate the dependence of amino acid occurrence between two positions, *A*_i_ and *A*_j_ that surround the ubiquitylated substrate sites. In order to extract motifs that have conserved biochemical properties, MDD categorizes the 20 types of amino acids into five groups such as aliphatic, polar and uncharged, acid, basic and aromatic groups. The MDD clustering is a recursive process that divides the data sets into tree-like subgroups. The MDD terminates after all of the subgroup sizes are less than the value of the specified value of maximum-cluster-size. In this investigation, MDD was utilized to subdivide 2486 non-homologous ubiquitylation sites into nine subgroups that contain significant substrate motifs. Finally, WebLogo (64, 65) was adopted to generate the graphical sequence logo for the relative frequency of the corresponding amino acid at each position around the ubiquitylation sites.

## Results and Discussion

### Data content in the database of UbiNet

After manually reviewing over 600 research articles (dated up to 1 July 2015) obtained from a text mining method, a total of 43 948 ubiquitylation sites were obtained from 14 692 ubiquitylated human proteins supported by 464 literatures. Redundant data among heterogeneous online resources were removed, and the remaining data contained two E1 activating enzymes, 46 E2 conjugating enzymes and 499 E3 ubiquitin ligases that were experimentally confirmed in humans. There is currently very limited information regarding which E3 ligase is responsible for the ubiquitylation of a specific substrate protein. Hence, we retrieved the physical PPIs between E3 ligases and ubiquitylated proteins to infer the potential E3 ligase for regulating an ubiquitylated protein. As presented in Table 1, a total of 10 437 physical PPIs between 438 E3 ligases and 2839 ubiquitylated proteins were obtained. Particularly, 29 257 PPIs between E3 ligases and other proteins could be used to discover potential substrates for E3 ligases, by combining the ubiquitylation site prediction method with MDD-identified substrate motifs (56). In addition, 413 054 PPIs appeared to be associated with ubiquitylated proteins, which could be used to study their functional associations on the basis of their interactions with each other. In this investigation, all of the data used in the construction of protein ubiquitylation networks were experimentally verified and supported with 44 184 research articles.

**Table 1.** Data content in UbiNet

Data content	Number of records
Ubiquitylated protein (potential E3 substrates)	14 692
Ubiquitylation sites	43 948
Number of articles supporting ubiquitylation data	464
E1 activating enzymes	2
E2 conjugating enzymes	46
E3 ubiquitin ligases	499
PPIs between E3 ligases and other proteins	29 257
PPIs between ubiquitylated proteins and other proteins	413 054
PPIs between E3 ligases and ubiquitylated proteins	10 437
E3 ligases interacting with ubiquitylated proteins	438
Ubiquitylated proteins interacting with E3 ligases	2839
Supported articles	44 184

### Functional associations of 499 E3 ubiquitin ligases in humans

Based on the InterPro annotation of protein domains, Table 2 shows that the most abundant protein domain within E3 ubiquitin ligases appeared to be the ‘WD40 repeat’, whose major function is to mediate interaction between the E3 ligase and its substrates (66). Another abundant protein domain is the ‘Zinc finger, RING-type’, which could bring the E2 enzyme and substrate together and mediate the transfer of ubiquitin from E2 enzymes to the substrates (67). A RING finger domain is a structural domain of the zinc finger type that contains a Cys_3_HisCys_4_ amino acid motif binding two zinc cations. As presented in Supplementary Figure S3A, the RING finger domain coordinates the binding of Zn^2+ ^through specifically spaced cysteine and histidine residues (67). Instead of forming a catalytic intermediate with ubiquitin like the HECT domain, the RING finger domain acts as a scaffold that brings E2 and substrate together, and mediates the transfer of ubiquitin from E2 to the substrate (16, 68). As illustrated in Supplementary Figure S3B, the members of RING-type E3s can function as monomers, dimers or multi-subunit complexes, which are responsible for both binding to the E2 and stimulating ubiquitin transfer (69, 70).

**Table 2.** Distribution of the top 20 functional domains for 499 human E3 ligases

No.	InterPro ID	Domain terms	Number of proteins	% Total
1	IPR001680	WD40 repeat	289	57.9158%
2	IPR001841	Zinc finger, RING-type	64	12.8256%
3	IPR000315	Zinc finger, B-box	58	11.6232%
4	IPR006652	Kelch repeat type 1	58	11.6232%
5	IPR000408	Regulator of chromosome condensation, RCC1	55	11.0220%
6	IPR000569	HECT	39	7.8156%
7	IPR003877	SPla/RYanodine receptor SPRY	38	7.6152%
8	IPR002867	Zinc finger, C6HC-type	29	5.8116%
9	IPR018957	Zinc finger, C3HC4 RING-type	28	5.6112%
10	IPR013069	BTB/POZ	28	5.6112%
11	IPR001202	WW/Rsp5/WWP	28	5.6112%
12	IPR020683	Ankyrin repeat-containing domain	27	5.4108%
13	IPR001496	SOCS protein, C-terminal	25	5.0100%
14	IPR000571	Zinc finger, CCCH-type	22	4.4088%
15	IPR001876	Zinc finger, RanBP2-type	22	4.4088%
16	IPR011016	Zinc finger, RING-CH-type	22	4.4088%
17	IPR001258	NHL repeat	21	4.2084%
18	IPR011705	BTB/Kelch-associated	15	3.0060%
19	IPR002110	Ankyrin repeat	14	2.8056%
20	IPR001452	Src homology-3 domain	10	2.0040%

On the other hand, the HECT domain of E3 ligases plays prominent roles in trafficking, immune response and several other signaling pathways that regulate cellular growth and proliferation (71). The conserved HECT domain comprises ∼350 amino acids. As illustrated in Supplementary Figure S4a, the HECT domain consists of two major components: a N-terminal N-lobe that interacts with the E2 enzyme, and a C-terminal C-lobe which contains the active-site cysteine that forms the thioester linkage with ubiquitin (67, 72, 73). The conserved HECT domain is located at the C-terminus of these enzymes, whereas their N-terminal domains are diverse and mediate substrate targeting. Studies of the HECT domains and their crystal structures suggested that these two lobes (N- and C-lobe) are connected via a flexible hinge that allows them to come together during ubiquitin transfer (16). As shown in Supplementary Figure S4b, the HECT E3 ligases can catalyse two reactions. The first one is transesterification reaction, in which ubiquitin is transferred from the cysteine at the E2 active site to a cysteine in the HECT domain; the second reaction is the subsequent attack on the ubiquitin-binding HECT thioester by a substrate lysine (74). Moreover, based on the functional annotations from the GO database, distributions of the 499 E3 ligases in the context of biological processes, molecular functions and cellular components are presented in Supplementary Table S3. Investigation of these biological process indicated that most E3 ligases appeared to be associated with the regulation of cellular processes. Additionally, investigation of the molecular functions revealed that E3 ligases may be mostly involved in the binding of proteins, enzymes and transcription factors.

### Functional associations of 14 692 non-redundant ubiquitylated proteins in humans

With respect to the functions of 14 692 ubiquitin-conjugated human proteins, ∼70% of the collected 43 948 ubiquitylation sites appeared to be located within specific functional domains, adding support to the biological importance of ubiquitylation. Supplementary Table S4 presents the top 50 InterPro functional domains harboring ubiquitylated sites in humans. This investigation revealed that most ubiquitylation sites could be found within protein domains such as the histocompatibility complex (MHC) class I (alpha chain), Immunoglobulin (Ig) C1-set, WD40 repeat and Spectrin repeat. Ubiquitylation of the MHC class I domain has been reported in the context of viral proteins which target MHC class I protein for degradation in the endoplasmic reticulum and at the cell surface (75). Ig C1-set domains, which are classical Ig-like domains that resemble the antibody constant domain, were found exclusively in molecules involved in the regulation of immune responses, such as the MHC class I and II complexes, as well as various T-cell receptors (76). The WD40 repeat domain is present in proteins essential for many biological activities, including members of the F box family of SCF ubiquitin E3 ligase adaptors (77). The fact that this domain was found to be enriched among ubiquitylated sites may explain how ubiquitylation modulates a broad spectrum of cellular processes. Additionally, the distributions of GO biological processes, molecular functions and cellular components of 14 692 non-redundant ubiquitylated proteins are presented in Supplementary Table S5.

### Substrate motifs of ubiquitin-conjugation sites in humans

**Figure 2.**
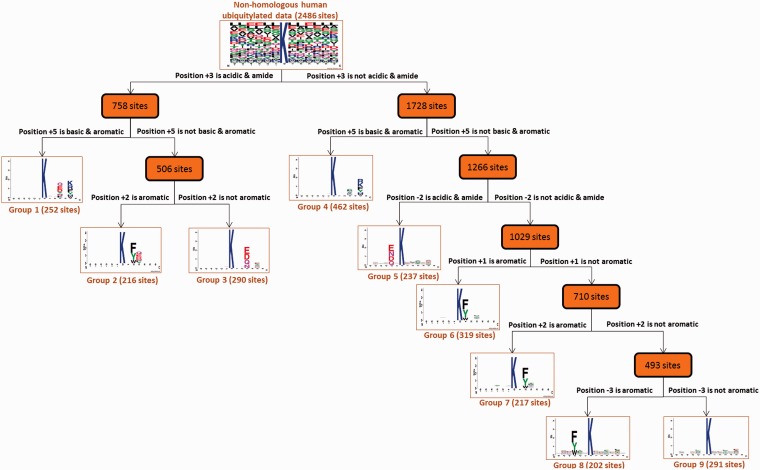
Tree view of MDD-clustered subgroups with statistically significant motifs for 2486 non-homologous human ubiquitylation sites.

As presented in Supplementary Figure S5, analysis of the position-specific amino aicd composition surrounding the ubiquitylated lysines indicated that Leu (L), Glu (E) and Ala (A) were the most conserved amino acid residues. This is consistent with a proteomic analysis of ubiquitylation site patterns in murine tissues (78). In order to obtain an unbiased motif analysis, the homologous sequences should be removed from all 43 948 ubiquitylation sites by using Cluster Database at High Identity with Tolerance (CD-HIT) (79) program with 30% sequence identity. Based on the 13-mer window length, 2486 non-homologous ubiquitylated sequences were obtained for the discovery of substrate motifs. To discover the conserved motifs from large-scale ubiquitylation data, the MDD clustering method was applied to explore the potential substrate motifs from non-homologous ubiquitylation sites. As shown in Figure 2, a total of nine MDD-identified subgroups containing conserved motifs were obtained from 2486 non-homologous human ubiquitylation sites. Group 1, which was composed of 252 ubiquitylation sites, contained the conserved amino acid composition at positions +3 and +5. In addition, for Groups 1–3, the conserved motif of acidic and amide residues were found at position +3. Group 2 harbored the conserved motif of Phenylalanine (F), Tyrosine (Y) and Trytophan (W) residues at position +2. On the other hand, the conserved motifs of Glutamic acid (E), Aspartic acid (D), Glutamine (Q) and Asparagine (N) residues were found at positions +3 and −2 in Groups 3 and 5, respectively. Overall, most of the groups contained the conserved motifs of Phenylalanine (F), Tyrosine (Y) and Trytophan (W) residues at various specific positions. This motif analysis suggested that, for human protein ubiquitylation, the substrate sites may be associated with the conserved motif of aromatic amino acids. In this investigation, if an interaction is identified between a protein and an E3 ubiquitin ligase, the MDD-identified substrate motifs could be adopted to discover the putative ubiquitylation sites with the corresponding conserved motif.

### E3-interacting motifs on 14 692 ubiquitylated proteins

The enzymes involved in the ubiquitylation process are known to play a variety of roles. For example, E2 conjugating enzymes can catalyse the transfer of ubiquitin from E1 to the cysteine within the active site of E2 via a trans(thio)esterification (1). Typically, ubiquitin conjugation at a target protein is carried out through three main steps. First, the C-terminus of ubiquitin is coupled to a reactive cysteine residue on the E1 activating enzyme via a thioester linkage. The activated ubiquitin is then transferred to an E2 conjugating enzyme, also via a thioester linkage. Finally, the E2-Ub complex bound to an E3 ligase recognizes the substrate and catalyzes the transfer of ubiquitin to a lysine residue on the substrate, forming an isopeptide linkage between the ubiquitin C-terminus and the ε-amino group of the lysine residue. Consequently, in contrast to E2 conjugating enzymes, E3 ligases do not usually bind or come in direct contact with the ubiquitylated sites. In order to uncover E3-interacting motifs, a motif discovery tool, MEME (80), was applied to discover potential motifs that might be recognized by E3 enzymes among ubiquitylated proteins known to interact with a specific E3 ligase. For instance, as provided in Supplementary Table S6, the breast cancer type 1 susceptibility protein (BRCA1), an E3 ubiquitin ligase (81, 82), contains a potential interacting motif [LGGxxFD] that was found to be present in 15 ubiquitylated substrates, as filtered from 170 BRCA1-interacting ubiquitylated proteins by MEME (*E*-value < 0.01). Based on the parameter setting of *E*-value < 0.01, a total of 38 potential motifs, existing in at least five ubiquitylated proteins, were obtained from 4770 ubiquitylated proteins that interact with E3 ligases.

### Web interface and utility of UbiNet

**Figure 3.**
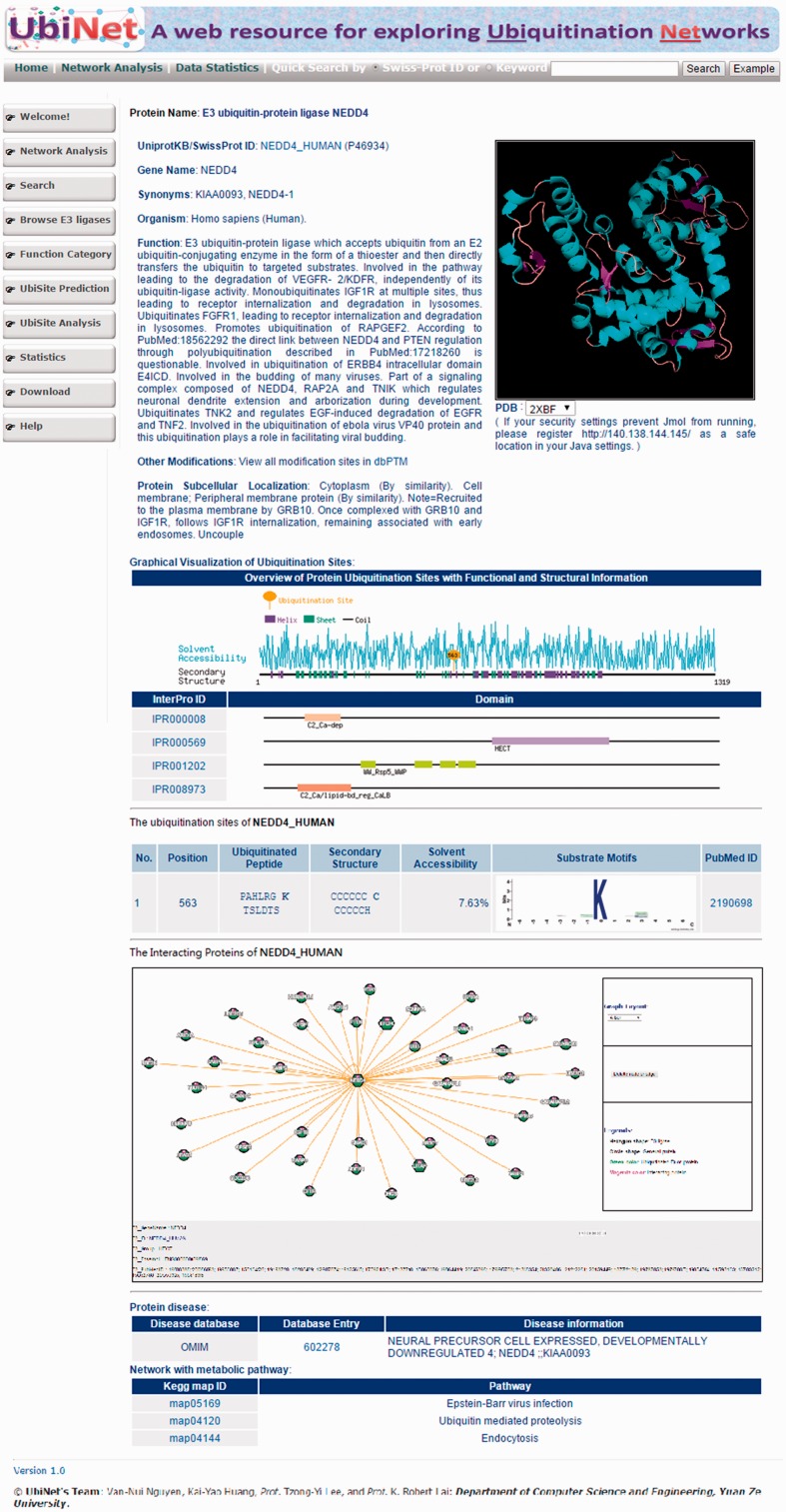
Web interface of a typical UbiNet query, including basic protein information, graphical visualizations of ubiquitylation sites with structural characteristics and functional domains, a table of ubiquitylated sites with substrate motifs and supported literature, a dynamic visualization of E3-substrate networks, disease associations and KEGG metabolic pathways associated with the network.

To facilitate the use of UbiNet, a web interface was designed for users to efficiently search for the proteins of their interest. Figure 3 shows the output of a typical UbiNet query, including basic protein information, graphical visualizations of the ubiquitylation sites with their structural characteristics and functional domains, a table of ubiquitylated sites with the corresponding substrate motifs and the supporting literature, dynamic visualizations of the E3-substrate networks, disease associations and KEGG metabolic pathways associated with the constructed network. As an example**,** given 21 proteins, UbiNet not only identified four E3 ligases and 14 ubiquitylated proteins, but it also revealed that three proteins interacting with two of the E3 ligases may be the potential ubiquitylated substrates (Supplementary Figure S6**)**. This example shows that Forkhead box protein O3 (FOXO3) might be the ubiquitylated substrate recognized by E3 ligase MDM2. Users can click on the protein FOXO3 and UbiNet will provide the predicted ubiquitylation sites along with their corresponding substrate motifs.

Another case study demonstrates the construction of a protein ubiquitylation network when the input contained only three E3 ligases (NEDD4, STUB1 and PELI2). As illustrated in Supplementary Figure S7, UbiNet could identify the potential substrates for the input E3 ligases based on PPI information and protein ubiquitylation data. For a specific E3 ligase, the interacting ubiquitylated proteins could be used to explore the functional associations among the proteins and build an ubiquitin regulatory network. A summary table of all 499 E3 ligases and ubiquitylated proteins that they interact with is provided on UbiNet. As an example given in Supplementary Figure S8, NEDD4L (E3 ubiquitin-protein ligase NEDD4-like) interacts with 17 ubiquitylated proteins, which can be visualized in the UbiNet network viewer.

**Figure 4.**
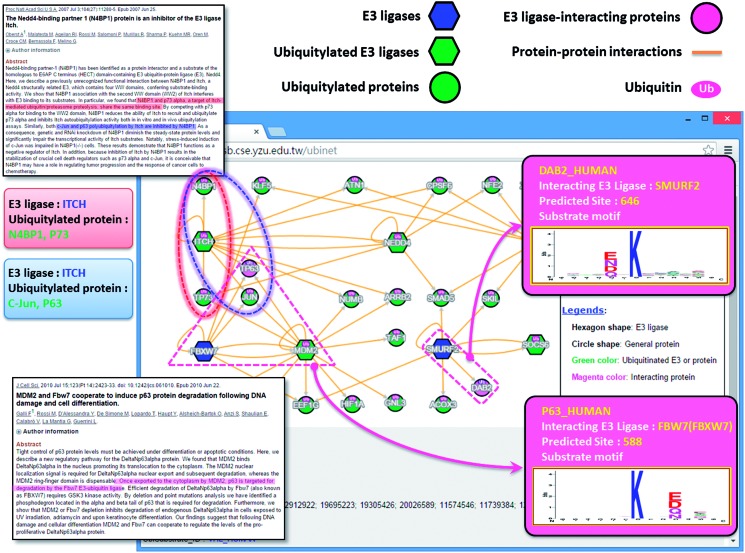
A case study exploring the regulatory network between E3 ubiquitin ligases and ubiquitylated substrates.

In addition to providing the potential substrates for an E3 ligase, UbiNet can identify the E3 ligases for a specific ubiquitylated protein without the annotation of its ubiquitylation enzymes. As demonstrated in another case study (Figure 4**)**, UbiNet is able to show that MDM2, a RING oncoprotein, can ubiquitylate tumor protein p73 (TP73) (83), which is a member of the tumor suppressor p53 protein family that can induce cell cycle arrest or apoptosis through the activation of p53-responsive genes, as well as p53-independent pathways (84). Although MDM2 is an established negative regulator of p53 (19), our case study results suggest that MDM2 might also be implicated in the regulation of p73 by ubiquitylation. This investigation also found that MDM2 can cooperate with Fbw7 ubiquitin ligase (FBXW7) to induce the degradation of TP63 following DNA damage and cell differentiation (85). Moreover, another E3 ubiquitin ligase, ITCH, was also found to regulate tumor proteins p63 and p73 (86) through protein ubiquitylation. In fact, UbiNet could be used to discover not only potential substrates for a specific E3 ligase, but also potential E3 ligases for ubiquitylated proteins. In addition, Figure 4 shows that SMURF2, an E3 ubiquitin ligase, may ubiquitylate disabled homolog 2 (DAB2) at position 646 based on the Group 5 substrate motif as identified by MDD (Figure 2**)**. Moreover, Figure 4 demonstrates that Fbw7 ubiquitin ligase (FBXW7) may be able to recognize TP63 as a substrate for ubiquitylation, which is consistent with a previous study (85).

## Conclusion

Motivated by the importance of protein ubiquitylation in the regulation of various biological processes, we developed an interactive viewer to provide a comprehensive map of intracellular ubiquitylation networks by integrating a variety of information regarding E3 ligases, ubiquitylated proteins and PPIs. The UbiNet database offers users an effective platform to efficiently study protein ubiquitylation networks among large-scale ubiquitylation data. The current version of UbiNet was designed specifically for humans to serve as not only a meaningful framework for E3-substrate regulatory networks but also a new scheme to discover potential E3 ligases for ubiquitylated substrates. The UbiNet system is now freely accessible via http://csb.cse.yzu.edu.tw/UbiNet/. In the future, improvement on UbiNet is expected as more data related to E1 activating enzymes, E2 conjugating enzymes and E3 ubiquitin ligases become available. To provide more adequate information needed for functional analysis, more detailed descriptions associated with the biological functions of ubiquitylation sites will be extracted from research articles by using an enhanced information retrieval system. A recent study (44) has extracted the spatial amino acid compositions from experimentally verified phosphorylation sites with 3D structures available in PDB. By adopting a similar strategy, we can envision that UbiNet can be greatly improved in prospective works by applying structural motif information to characterize ubiquitylation sites based on protein tertiary structures.

## Availability

The data content in UbiNet will be maintained and updated quarterly by continued surveys and reviews of public resources and research articles. This resource is now freely accessible online at http://csb.cse.yzu.edu.tw/UbiNet/. Experimentally verified ubiquitylation data could be downloaded as text files. Supplementary Figures S1–S8 and Tables S1–S6 are also available at DATABASE online.

## Supplementary data

Supplementary data are available at Database Online.

## Author’s contributions

T.Y.L. and K.R.L. conceived and supervised the project. V.N.N. and K.Y.H. were responsible for the project design and computational analyses. V.N.N. and T.Y.L. drafted the article with revisions by J.T.Y.W. All authors read and approved the final article.

*Conflict of interest*. None declared.
